# Pregnant under the pressure of a pandemic: a large-scale longitudinal survey before and during the COVID-19 outbreak

**DOI:** 10.1093/eurpub/ckaa223

**Published:** 2020-11-24

**Authors:** Elin Naurin, Elias Markstedt, Dietlind Stolle, Daniel Enström, Anton Wallin, Ingrid Andreasson, Birgitta Attebo, Ottilia Eriksson, Klara Martinsson, Helen Elden, Karolina Linden, Verena Sengpiel

**Affiliations:** 1 Department of Political Science, University of Gothenburg, Gothenburg, Sweden; 2 Society, Opinion and Media Institute, University of Gothenburg, Gothenburg, Sweden; 3 Department of Political Science, McGill University, Montreal, Canada; 4 Institute of Health and Care Sciences, Sahlgrenska Academy, University of Gothenburg, Gothenburg, Sweden; 5 Sahlgrenska University Hospital, Gothenburg, Sweden; 6 Department of Obstetrics and Gynecology, Sahlgrenska Academy, University of Gothenburg, Gothenburg, Sweden

## Abstract

**Background:**

One of the groups that is most vulnerable to the COVID-19 pandemic is pregnant women. They cannot choose to refrain from care; they and their children are at risk of severe complications related to the virus; and they lose comfort and support as clinics prohibit their partners and as societal restrictions demand isolation from friends and relatives. It is urgent to study how this group is faring during the pandemic and we focus here on their health-related worries.

**Methods:**

A longitudinal survey at a Swedish hospital starting 6 months before (16 September 2019) and continuing during the COVID-19 outbreak (until 25 August 2020). A total of 6941 pregnant women and partners of diverse social backgrounds were recruited. Ninety-six percent of birth-giving women in the city take early ultrasounds where recruitment took place. Sixty-two percent of the women with an appointment and fifty-one percent of all partners gave consent to participate.

**Results:**

Pregnant women experienced dramatically increased worries for their own health, as well as for their partner’s and their child’s health in the beginning of the pandemic. The worries remained at higher than usual levels throughout the pandemic. Similar, but less dramatic changes were seen among partners.

**Conclusions:**

There is a need for heightened awareness of pregnant women’s and partners’ health-related worries as a consequence of the COVID-19 pandemic. Related feelings, such as anxiety, have been linked to adverse pregnancy outcome and might have long-term effects. The healthcare system needs to prepare for follow-up visits with these families.

## Introduction

In 2019, a novel coronavirus emerged and caused the global COVID-19-pandemic, which has led to a high death toll and unprecedented consequences for society and healthcare. It is known that levels of worry, sleeping disorders and depression increased in the general population due to the pandemic.^1^ However, little is published on how the worries and mental well-being of one of the most vulnerable and exposed groups, pregnant women, who cannot choose to delay or refrain from care and are at higher risk for severe disease, have changed due to the pandemic.

Research showed that earlier corona virus infections (SARS and MERS) during pregnancy are associated with a high incidence of maternal and neonatal mortality and morbidity,^2^ and initial case series and early population-based studies indicated increased maternal morbidity and mortality and adverse pregnancy outcomes due to COVID-19.^3^^,^^4^ When the last study participants answered in this study, it was known that COVID-19 is associated with increased risk for preterm birth and admittance of the newborn to the neonatal unit.^5^ In addition, women lose support and comfort during the pandemic, as clinics prohibit their partners from joining in maternal healthcare and societal restrictions demand social isolation from friends and relatives. It is thus feared that worry is increased among all pregnant women, and not only those with COVID-19.

Self-reported worry and the related feeling of anxiety during pregnancy are common even under normal circumstances.^6^^,^^7^ Worries related to the health of the unborn child are universal, spanning different populations.^8^^,^^9^ It is also known that anxiety and stressful life events are associated with adverse pregnancy outcomes, such as preterm birth, foetal growth restriction, low birth weight^10^^,^^11^ and children’s early negative reactivity and self-regulation.^12–15^ Some of these risks have been shown to be reduced by the presence of an involved partner^16–18^ and a sense of togetherness^19^ during the pregnancy.

A few studies have examined feelings of worry and anxiety among pregnant women during the COVID-19 pandemic. These have either been qualitative studies mapping few respondents^20–22^ or smaller scale surveys in single countries^21^^,^^23–25^ or convenience samples in several countries.^26^ All these indicate that the new coronavirus has the potential to increase worry in the pregnant population. However, very few studies had the possibility to follow a pregnant population from before the COVID-19 pandemic with the potential to measure actual changes. One study followed 200 Italian women and found significantly increased feelings of worry.^25^ Another followed 63 women in Turkey, finding significant increases of anxiety and depression symptoms.^27^ No study so far, focussed on the experience of partners of pregnant women. The aim of this study is to determine the effect of the COVID-19 pandemic on the worries of a large and varied population of pregnant women and their partners regarding their own health, that of their partner, and unborn child in Sweden, a country with free maternal care and high trust in the welfare state. It also aims to determine which groups are especially susceptible to heightened levels of health-related worries during the pandemic.

## Methods

### Study design

This study is based on a large-scale longitudinal survey of pregnant women and their partners before and during the COVID-19 pandemic, the Swedish Pregnancy Panel. Recruitment of participants started in September 2019 in the ultrasound clinic waiting area at Östra Sahlgrenska University Hospital in Gothenburg, Sweden. Sweden offers free antenatal care to all pregnant women, and almost all women undergo routine ultrasound scans during the first and second trimester. Östra Sahlgrenska University Hospital is a tertiary care unit with about 10 000 deliveries/year,^28^ and it is the only hospital with a delivery unit in the area of Gothenburg, which includes 1040 000 inhabitants. The hospital is a uniquely well-suited place to recruit women and partners in early pregnancy, as 96% of all the routine second trimester ultrasound (around pregnancy week 19) in the city area are performed there. Furthermore, around 55% of all first trimester ultrasounds (around pregnancy week 12) are performed at the hospital. This means that 96% of all pregnant women in the area visited the ultrasound clinic’s waiting area, and that around half of these did so around pregnancy week 12, and more or less all did so around pregnancy week 19.

### Participants and enrolment

Pregnant women (and their partners) were eligible to participate if they were in pregnancy weeks 12–19 and could understand oral and written information about the study. A partner is defined as a person who is married or living in partnership together with or apart from the pregnant person or is the biological or non-biological parent of the child/children to be born. In practice, the partner of the pregnant woman was almost always in the waiting room during recruitment of participants. No other specific inclusion or exclusion criteria were used. Of the partners, 2.3% were women.

### Questionnaires

Eligible participants were given information about the study and they provided written consent before filling in a background questionnaire on a tablet and logging their email address. Questionnaires were then sent out electronically during pregnancy weeks 12–19, 22–24 and 36. Questionnaires were available in Swedish, English, Arabic and Somali. Recruitment had to be stopped on 18 March 2020, since only patients and healthcare staff were allowed to enter the hospital area at that time due to the spread of COVID-19. However, those who had already been recruited continued to participate online. Participants gave consent for three follow-up questionnaires after pregnancy; when the baby is 2 months, 1 year and 2 years. These follow-up questionnaires were not included in this article since they were still ongoing at the time of analysis.

### Outcomes

The questionnaires were developed in a multi-disciplinary research collaboration between political scientists, obstetricians and midwives. One of the aims was to clarify the impact of pregnancy, childbirth and early parenthood on individuals’ health-related worries. Health-related worries are reported using three items: ‘How worried are you currently about the following?’: ‘Your own health’, ‘The health of the unborn’, ‘Your partner’s health’. Participants answered on a seven-point Likert scale ranging from ‘1 Not worried at all’ to ‘7 Very worried’. In previous work on women’s self-reported feelings of worry in Sweden, the term ‘worry’ (‘oro’), was used as an operational definition of anxiety.^29^^,^^30^ However, it is notable that our measures are not validated scales for the medical term of anxiety, which is a concept often used in the literature we cite above.^31^

Views of the coronavirus and its consequences are here reported in three ways. First, the Swedish Pregnancy Panel offered several possibilities for the respondents to use their own words in open-ended survey responses. A keyword analysis of these responses was conducted to determine the frequency of mentions of ‘corona’, ‘COVID’, ‘SARS’, ‘pandemic’ and ‘virus’. This enabled detection of when participants started to spontaneously mention the virus. Second, respondents were asked ‘How often do you think about the coronavirus and its consequences?’ Answer options were: ‘1 Never or almost never’, ‘2 Very rarely’, ‘3 Quite rarely’, ‘4 Sometimes’, ‘5 Quite often’, ‘6 Very often’ and ‘7 All the time, or almost all the time’. Those responses are compared with ‘How often do you think about the pregnancy and the forthcoming childbirth,’ with the same answer options. Third, the respondents were asked to describe how they are personally affected by the coronavirus and its consequences. Answers to this open-ended item were grouped into 47 inductively coded categories, which were collapsed into nine broader themes capturing the content of the answers and following common methodological practices^32^ (details are found in a comment under figure 2).

In Supplementary figure S1, additional outcome variables are reported, e.g. the overtime developments for: ‘In general, how much confidence do you have in the following institutions and actors in Sweden?’ ‘Healthcare’; ‘Politicians’ and: ‘How satisfied are you with the following types of care that you and your family received in connection with the pregnancy?’ ‘Planned prenatal care during the pregnancy’; ‘Emergency care during the pregnancy’. In some analyses, Age, Education, Income, Immigrant background and Time spent with friends, are used as additional background variables, and are then described in figure legends. Descriptive statistics and traits of all variables included in the article are found in Supplementary table S1.

### Analytical strategy

We follow pregnant women and partners over time before and during the COVID-19 pandemic between September 2019 and August 2020. For analyses of differences over time we used monthly estimates based on weighted pooled ordinary least squares estimates, controlling for pregnancy stage. To illustrate statistical significance, we employ a pre–post design using March 12 as a benchmark to help define whether the outcomes are significantly different before and during the pandemic. 12 March is the date when partners were prohibited at ultrasound clinics, maternity wards, and other planned appointments at the hospital. The date provides a reasonable estimate of before and during the pandemic for the participants recruited at the targeted hospital. The date also coincides with other major developments in the COVID-19 pandemic. On 11 March, the World Health Organization characterized COVID-19 as a pandemic and this was also the date of the first domestic death of COVID-19 in Sweden. A few days later, on 19 March, the participants in the Swedish Pregnancy Panel/learned that partners were no longer allowed in maternity clinics in the city. We performed robustness tests using dates before and after 12 March and those did not alter our conclusions.

As estimates at any given time point are pooled across different questionnaires, it is possible that the relative distribution between questionnaires reflects factors related to certain stages of pregnancy. Although we control for fixed questionnaire effects, unobserved heterogeneity related to pregnancy may be unaccounted for. Therefore, we ran separate analyses by questionnaire, without altering our conclusions. We use the full unbalanced sample in all analyses. This means that we do not limit the set of respondents to those who have answered all questionnaires. To provide robustness checks, we tested varying model specifications, such as a balanced sample and alternative estimators, including random effects and fixed effect models with similar overall results (see Supplementary table S2).

For comparability across our outcomes, the variables are normalized to a 0–1 range. Normalized coefficients can be interpreted in terms of percentages of the full range of the scale. As we are using unbalanced panel data, we counter the nonresponse bias by using inverse probability weights. The weights are based on logistic models where response propensities are estimated for each questionnaire and gender, education, age, and interest in politics and societal issues. All significance tests were two-sided at the 95% significance level. Statistical analyses were performed using Stata version 16.

### Ethics

The Swedish Ethical Review Authority approved the study on 15 April 2019 (Dnr: 1061-18).

## Results

### Descriptives

During the recruitment period, 6133 pregnant women had a first and/or second trimester ultrasound at Östra Sahlgrenska University Hospital, 3,828 of whom gave consent to participate in the Swedish Pregnancy Panel (62%). As 1% of the pregnant women told us that they did not have a partner, we estimated that our total partner population was 6045. Of these, 3113, gave consent to participate (51%). In Supplementary tables S1 and S4, we give baseline characteristics and a description of the loss of participants over time.

### Views about the coronavirus and its consequences

**Figure 1:**
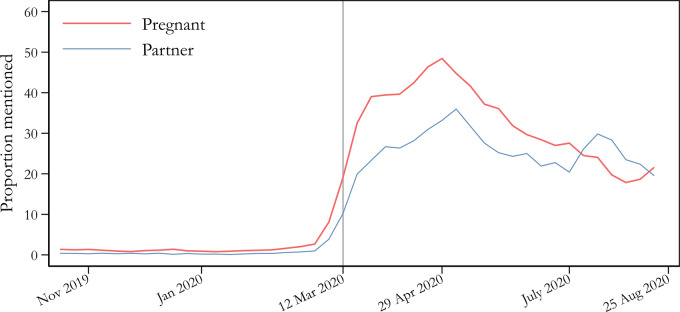
Mentions of ‘COVID’, ‘corona’ and related keywords in the Swedish Pregnancy Panel during late 2019 and first half of 2020. Notes: The proportion is calculated based on the total number of answers in the different questionnaires. The base of the calculation is thus the number of answers that potentially could include COVID-19-related answers on 1 single day, here illustrated using a 3-week moving average of the observed percentage of survey responses that mentioned at least one of the keywords ‘COVID’, ‘corona’, ‘SARS’, ‘pandemic’ or ‘virus’ for any of the open-ended questions in any of the active surveys. The average proportion among pregnant women before 12 March was 1.6% (*n* = 6893) and post was 35.7% (*n* = 2498). The corresponding numbers for partners are 0.6% (*n* = 6562) and 26.0% (*n* = 1625). Among the 12 open answer questions were questions about important societal issues, feelings evoked by pregnancy, what the respondents discuss with friends, and general comments at the end of the survey.

Figure 1 shows the mentions of COVID-19-related keywords by pregnant women and their partners. The y-axis in figure 1 indicates the proportion of responses that include at least one such keyword in the open answer items. Respondents started to mention the pandemic in open answer items often in mid-March. The proportion of spontaneous mentions by pregnant women rose from 0 to the peak at 48%, and the frequency for partners rose from 0 to around 36%. Between 12 March and 25 August, about a third of all open responses from pregnant women (32%) mentioned the virus in one form or another (25% for partners), and the attention paid to this topic persisted throughout the study period. It is hard to imagine any other subject that would be so often spontaneously mentioned over such a long period of time. As a comparison, we looked at three other types of keywords that were connected to highly salient events and issues during the study period, namely the UN climate action meeting and Greta Thunberg’s journey across the Atlantic early fall 2019; the presentation of a study performed at the hospital where our recruitment took place that indicated higher risk of death of child with induction after rather than during pregnancy week 41^33^; as well as keywords connected to immigration and crime, which were seen as ‘the most important societal problem’, according to several population-based opinion surveys during fall 2019.^34^ None of these received as high levels of spontaneous mentions as the COVID-19 pandemic (data not shown).

On the direct question of how often they think about the coronavirus and its consequences, 89% of the pregnant women answered that they think about it ‘fairly often’, ‘very often’ or ‘all the time, or almost all the time’ on average between 24 March and 25 August. The same number for the partners was 83%. This can be compared with how often they said they thought about ‘pregnancy and the forthcoming birth’ during the same time, which was 91% of the pregnant women and 79% of the partners. Although the pregnant women and (to a somewhat lesser degree) their partners thought about pregnancy and the forthcoming birth often, the virus occupied their thoughts to about the same degree.

**Figure 2:**
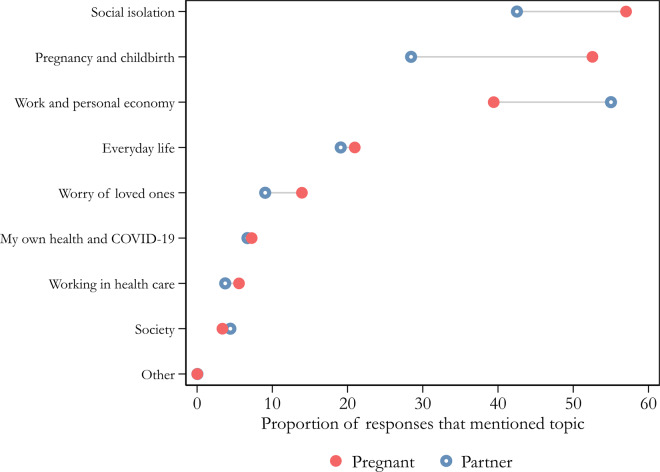
Ways in which respondents are personally affected by the coronavirus and its consequences. Notes: Unweighted estimates. Answers given between 24 March and 25 August, 2020. Pooled N, pregnant woman = 4576; partner = 2080 (individual *n*, pregnant woman = 1723, partner = 1016). Question wording: ‘In what way are you personally affected by the coronavirus and its consequences?’ Examples of codes used for the reported categories: Work and personal economy: Changed workload, Lost savings, I lost my job. Social isolation: Have chosen/been forced to isolate myself completely or partly, Do not see friends, Grandparents not allowed to meet the newborn baby. Pregnancy and childbirth: Worry about the childbirth, Worry about deficiencies in healthcare, Worry about the foetus’/newborn’s health, Partner cannot be present at the time of childbirth. Everyday life: Public transportations, Acting according to authorities’ advices, Helping others with purchases etc, Cancelled events, activities and trips. Society: Media reporting, Critical towards how society deals with the crisis, The impact on the economy on the societal level.

Figure 2 shows the results for the open answer item that asked participants to describe how they are personally affected by the coronavirus and its consequences. Pregnant women and their partners regard pregnancy and childbirth as directly related to the pandemic. Among the pregnant women, ‘pregnancy and childbirth’ was the category that was the second most often mentioned (53%), preceded only by ‘social isolation’, which was mentioned by as many as 57%. Among partners, ‘pregnancy and childbirth’ was the third most often mentioned category (28%), after ‘work and personal economy’ (55%) and ‘social isolation’ (43%). About two thirds of the answers in the ‘pregnancy and childbirth’ category can be distributed into subcategories that are connected to the exclusion of partners from maternal healthcare, including (i) antenatal clinic appointments, (ii) parental education, (iii) the birth of the child and (iv) the maternity ward. Jointly, these four subcategories were mentioned by 30% of the pregnant women and 32% of the partners. Thus, partners’ exclusion from maternal care clearly ranks higher than the ‘worry about loved ones’ and impact on ‘society’ categories when participants describe how they are personally affected by the pandemic.

### Respondents’ worry about their own health that of the child and that of their partner

**Figure 3:**
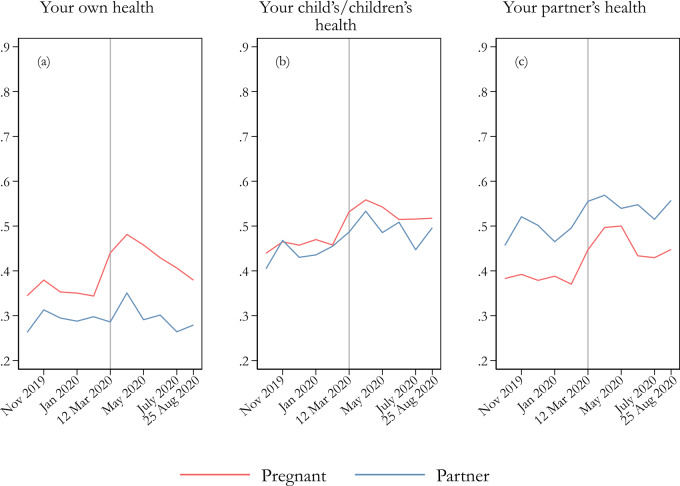
Worry about one’s own health (a), the child’s health, and (c) the partner’s health. Notes: Weighted estimates of a normalized dependent variable (ranging 0–1). Pooled *n*, (a) = 7701 (4410 individuals); (b) = 7304 (4293 individuals); (c) = 7800 (4373 individuals). Question wording: ‘How worried are you currently about the following?’; eight response alternatives: 1 ‘Not at all worried’ to 7 ‘Very worried’, and 8 ‘Not applicable’. The opt-out alternative was not included in the analysis (0.2%, 5.0% and 1.0%, respectively, of the overall responses for the three items reported).

Figure 3 illustrates how health-related worries have changed compared with before the COVID-19 outbreak through a long time series of items. Overall, there is a clear shift in health-related worries during the outbreak, with a strong rise observed among pregnant women, especially in mid-March 2020. Partners also experience increased worry, but to a somewhat lesser degree. Supplementary table S3 shows the results of tests examining whether changes are significant for the pre/post-12 March period. All types of health-related worry increased significantly during the COVID-19 pandemic, except the partners’ worry about their own health. In fact, the pregnant women’s worry about their own health increased by as much as 9.6 percentage points on average. Put differently, this means that pregnant women answer, on average, around half of a scale point higher on a seven-point scale for the pre/post-12 March period and considerable higher in the beginning of the pandemic. To this, they add their increased worry for the child’s and the partner’s health, both of which show similar increases.

**Figure 4:**
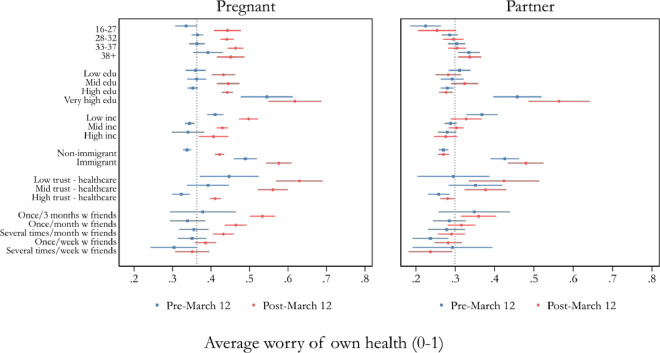
Respondents’ pre- and post-12 March worry for their own health by subgroup. Notes: The visualized means are the pooled unweighted worry levels before and after 12 March (normalized to range from 0 to 1). 95% CIs are shown for each parameter. The vertical line displays the pooled means of the entire period for pregnant women (0.36) and partners (0.30), respectively. Education is divided into four categories, recoded from a nine-category question: Low education = up to ‘Upper secondary school’, mid-level education = up to ‘University/college education, shorter than 3 years’, high education = ‘University/college education, longer than 3 years’, very high education = ‘PhD’. Low income = up to 25 999 SEK/month (∼2800 USD), mid-level income: 26 000–44 999, high income: 45 000+. Healthcare trust question wording: ‘In general, how much confidence do you have in the following institutions and actors in Sweden?—Healthcare system’; Low trust = 1 ‘Very Little’ and 2 ‘Not much’, mid-level trust = 3 ‘Neither a lot nor a little’, high trust = 4 ‘Quite a lot’ and 5 ‘A lot’. Be with friends, question wording: ‘In the last three months, how often have you done the following: Spent time with friends’: Sometime during the last 3 months, About once a month, Several times a month, About once a week, Several times a week, Every day.

Figure 4 illustrates variation in participants’ worry for their own health between different groups. The vertical line displays the pooled means of the pre-12 March period for pregnant women (0.36) and partners (0.30), respectively. Worry levels are higher for those with low income (<26 000 SEK/month), those with an immigrant background and those who have very high levels of education (ongoing or finished PhD education). These patterns are present both before and during the pandemic, for both pregnant women and partners. During the pandemic, all women but those who met their friends often, increased their levels of worry above the pre-pandemic average. Among the partners, there are two groups that disperse from the average during the pandemic: those who rarely met friends and those who did not have high trust in healthcare.

## Discussion

The main result of this study is that pregnant women’s worry for their own health, that of their unborn child(ren), and that of their partner increased significantly during the COVID-19 pandemic. It also shows that partners’ worry about the pregnant women’s and unborn children’s health increased significantly. Furthermore, the COVID-19 pandemic is perceived as directly affecting the pregnancy and forthcoming birth, often exemplified by the fact that partners are excluded from maternal healthcare. These results apply to Sweden, a high-trust environment in which confidence in healthcare and politicians further increased during the pandemic (Supplementary figure S1). Some groups were more worried about their health than the average participant already before the pandemic: low income, immigrant background and very high education mattered among both pregnant women and partners, which supports previous work concluding that certain groups are especially exposed to feelings of worry during and after pregnancy.^35^ As the pandemic evolved, the only group of pregnant women who showed no increase in worry for own health were those who often met with friends. Among partners, the pandemic led to higher levels of worry for own health among those with low trust in healthcare and among those who rarely met with friends.

This study contributes to the understanding of how vulnerable citizens reacted to the challenges of a pandemic crisis. The findings report levels of health-related worry. In a wider sense, worry is a state of mind that is closely related to anxiety,^31^^,^^36^^,^^37^ which in turn is known to be related to both mental and physical health during pregnancy and early parenthood, relationships and parenting skills,^38^^,^^39^ multiple perinatal outcomes,^12^^,^^13^ children’s development^12–15^ and partners’ ability to support their pregnant partners.^40^

A strength of this study is its design, capturing a unique, diverse set of pregnant women and their partners before and during the COVID-19 pandemic with continuous measures over time and over different stages of pregnancy. To our knowledge it is also the only study reporting from a partner’s perspective. Another strength is the large and varied sample.

There were some potential limitations of the current research. First, the unavoidable pause in recruitment meant that we were unable to study pregnant women and partners in the first weeks of pregnancy during a later stage of the pandemic. Second, levels of worry likely differ between different contexts, and while our study reaches a broad sample, it is concentrated in a city area in Sweden. Finally, the study has not determined the long-term consequences of these increased worries for the child’s development and parents’ mental and physical health; this must be subject to further investigations.

Childbirth and becoming a parent are among the most important events in a person’s life, and they are naturally associated with a certain level of worry for the child’s and mother’s well-being. Yet, as evidenced by our results, worry levels soared after the onset of the pandemic, indicating additional stress on top of the normal pregnancy-related worry. Clustering all these factors that require change and life adjustment together (i.e. personal and societal changes), pregnancy during the pandemic may be a major stressful life event that increases the risk of impaired long-term health for women, new-borns and partners. This warrants extra clinical attention to this generation of pregnant women, children and partners in follow-up medical care visits.

Future research should look into how the changed routines and increased stress levels during pregnancy affect pregnancy outcome, bonding between parents and their children and possible long-term effects of the COVID-19 pandemic also among non-infected women and partners and their children. Furthermore, there is a need to map how and when changes in clinical practices were implemented during the COVID-19 pandemic and whether some were more or less effective in mitigating women’s and partner’s worry.

## Supplementary data

Supplementary data are available at *EURPUB* online.

## Funding

The Swedish Research Council (2015-01546), Knut and Alice Wallenberg Foundation (KAW2017.0245), the University of Gothenburg (UGOT) funded this research.

*Conflicts of interest:* None declared.

**Key points**

- Thanks to a longitudinal survey targeting a total sample of pregnant women and partners at a Swedish hospital with 10 000 deliveries/year that started 6 months before the outbreak of the COVID-19 pandemic, the text gives a unique understanding of the development and consequences of the pandemic from the view of pregnant women and their partners.
- Pregnant women experienced dramatically increased health-related worries during the pandemic in the form of worries for the own health, as well as for partner’s and child’s health.
- Our analysis implies that pregnancies and births during the COVID-19 pandemic will have higher than normal risks of worry-related health effects.
- Excessive worry and anxiety has been linked to adverse pregnancy outcome in previous studies and might have long-term effects on pregnant women, their children and partners.
- There is a need for routines to follow-up on pregnant women, their children and partners during the COVID-19 pandemic.

## Supplementary Material

ckaa223_Supplementary_Data
