# Supplementary material for: Pregnant under the pressure of a pandemic: a large-scale longitudinal survey before and during the COVID-19 outbreak
Source: Eur J Public Health. 2020 Nov 24;31(1):7–13. doi: 10.1093/eurpub/ckaa223 (PMC7717243; doi:10.1093/eurpub/ckaa223)
Supplement: ckaa223_Supplementary_Data [file ckaa223_supplementary_data.zip › Supplementary File EJPH revise_non-anonymized.docx]

Pregnant under the pressure of a pandemic: a large-scale longitudinal survey before and during the COVID-19 outbreak.

Elin Naurin, Elias Markstedt, Dietlind Stolle, Daniel Enström, Anton Wallin, Ingrid Andreasson, Birgitta Attebo, Ottilia Eriksson, Klara Martinsson, Helen Elden, Karolina Linden, and Verena Sengpiel.

*European Journal of Public Health. Online from November 2020.*

## Supplement formulations

The supplement includes four tables and one figure, showing descriptive statistics, sensitivity analyses for robustness for the changes in worry post-March 12, institutional trust in healthcare and politicians, as well as satisfaction with planned prenatal and emergency care during pregnancy. We also report the tests for all pre-post differences of worry and response statistics. Additional information is provided upon request.

Table S1. Descriptive statistics

|  |  |  | *Pregnant* |  |  | *Partner* |  |  | *Total* |  |  |
| --- | --- | --- | --- | --- | --- | --- | --- | --- | --- | --- | --- |
| *Dependent variable* | Ques-tion-naires* | Range | Mean | Md | n | Mean | Md | n | Mean | Md | n |
| Worry about – your own health | 2–3 | 1–7 | 3.43 | 3 | 4,752 | 2.82 | 2 | 3,054 | 3.19 | 3 | 7,806 |
| Worry about – your child's/children's health | 2–3 | 1–7 | 4.02 | 4 | 4,541 | 3.85 | 4 | 2,863 | 3.95 | 4 | 7,404 |
| Worry about – your partner's health | 2–3 | 1–7 | 3.56 | 3 | 4,676 | 4.15 | 4 | 3,056 | 3.79 | 4 | 7,732 |
| Institutional trust - Healthcare system | 0, 3 | 1–5 | 3.90 | 4 | 5,775 | 3.92 | 4 | 4,221 | 3.91 | 4 | 9,996 |
| Institutional trust - Politicians | 0, 3 | 1–5 | 2.85 | 3 | 5,732 | 2.61 | 3 | 4,204 | 2.75 | 3 | 9,936 |
| Maternal care satisfaction – planned prenatal care | 1–3 | 1–5 | 4.32 | 5 | 7,443 | 4.30 | 5 | 4,856 | 4.31 | 5 | 12,317 |
| Maternal care satisfaction – emergency care | 1–3 | 1–5 | 3.97 | 4 | 2,397 | 3.94 | 4 | 1,793 | 3.96 | 4 | 4,196 |
| Thinking about pregnancy and forthcoming birth | 1–3 | 1–7 | 5.67 | 6 | 7,465 | 5.19 | 5 | 4,984 | 5.47 | 6 | 12,467 |
| Thinking about the coronavirus | 1–3 | 1–7 | 5.57 | 6 | 2,145 | 5.34 | 6 | 1,395 | 5.48 | 6 | 3,544 |
|  |  |  |  |  |  |  |  |  |  |  |  |
| ***Background variables*** |  |  | **Pct** |  | **n** | **Pct** |  | **n** | **Pct** |  | **n** |
| Age |  |  |  |  |  |  |  |  |  |  |  |
| *16-27* |  |  | 19 |  | 653 | 12 |  | 326 | 15 |  | 979 |
| *28-32* |  |  | 45 |  | 1,582 | 36 |  | 1,026 | 41 |  | 2,607 |
| *33-37* |  |  | 28 |  | 968 | 33 |  | 936 | 30 |  | 1,904 |
| *38+* |  |  | 9 |  | 313 | 19 |  | 528 | 13 |  | 841 |
| *Total* |  |  | 100 |  | 3,516 | 100 |  | 2,816 | 100 |  | 6,331 |
|  |  |  |  |  |  |  |  |  |  |  |  |
| Educational attainment |  |  |  |  |  |  |  |  |  |  |  |
| *Low education* |  |  | 17 |  | 407 | 22 |  | 357 | 19 |  | 764 |
| *Mid education* |  |  | 18 |  | 421 | 19 |  | 296 | 18 |  | 717 |
| *High education* |  |  | 61 |  | 1,467 | 54 |  | 852 | 58 |  | 2,319 |
| *Very high education* |  |  | 4 |  | 92 | 5 |  | 86 | 4 |  | 178 |
| *Total* |  |  | 100 |  | 2,387 | 100 |  | 1,591 | 100 |  | 3,978 |
|  |  |  |  |  |  |  |  |  |  |  |  |
| Personal monthly income |  |  |  |  |  |  |  |  |  |  |  |
| *Low income* |  |  | 27 |  | 621 | 14 |  | 220 | 22 |  | 841 |
| *Mid income* |  |  | 65 |  | 1,526 | 62 |  | 974 | 64 |  | 2,500 |
| *High income* |  |  | 8 |  | 189 | 24 |  | 377 | 14 |  | 566 |
| *Total* |  |  | 100 |  | 2,336 | 100 |  | 1,571 | 100 |  | 3,907 |
|  |  |  |  |  |  |  |  |  |  |  |  |
| Immigrant background |  |  |  |  |  |  |  |  |  |  |  |
| *Non immigrant* |  |  | 82 |  | 1,965 | 83 |  | 1,324 | 83 |  | 3,289 |
| *Immigrant* |  |  | 18 |  | 417 | 17 |  | 268 | 17 |  | 685 |
| *Total* |  |  | 100 |  | 2,382 | 100 |  | 1,592 | 100 |  | 3,974 |
|  |  |  |  |  |  |  |  |  |  |  |  |
| Habits - Spent time with friends |  |  |  |  |  |  |  |  |  |  |  |
| *Once/3 months* |  |  | 9 |  | 231 | 9 |  | 171 | 9 |  | 402 |
| *Once/month* |  |  | 23 |  | 619 | 23 |  | 434 | 23 |  | 1,053 |
| *Several times/month* |  |  | 29 |  | 764 | 26 |  | 492 | 27 |  | 1,256 |
| *Once/week* |  |  | 29 |  | 769 | 30 |  | 572 | 29 |  | 1,341 |
| *Several times/week* |  |  | 11 |  | 293 | 13 |  | 239 | 12 |  | 532 |
| *Total* |  |  | 100 |  | 2,676 | 100 |  | 1,908 | 100 |  | 4,584 |
|  |  |  |  |  |  |  |  |  |  |  |  |
| Institutional trust - Healthcare system |  |  |  |  |  |  |  |  |  |  |  |
| *Low trust* |  |  | 8 |  | 282 | 8 |  | 233 | 8 |  | 515 |
| *Mid trust* |  |  | 15 |  | 518 | 16 |  | 448 | 15 |  | 966 |
| *High trust* |  |  | 77 |  | 2746 | 76 |  | 2177 | 77 |  | 4,923 |
| *Total* |  |  | 100 |  | 3546 | 100 |  | 2858 | 100 |  | 6,404 |
|  |  |  |  |  |  |  |  |  |  |  |  |
| Institutional trust - Politicians |  |  |  |  |  |  |  |  |  |  |  |
| *Low trust* |  |  | 35 |  | 1217 | 48 |  | 1363 | 41 |  | 2,580 |
| *Mid trust* |  |  | 45 |  | 1565 | 35 |  | 992 | 40 |  | 2,557 |
| *High trust* |  |  | 21 |  | 730 | 17 |  | 488 | 19 |  | 1,218 |
| *Total* |  |  | 100 |  | 3512 | 100 |  | 2843 | 100 |  | 6,355 |

Notes: Descriptive statistics of dependent and independent variables summarize the time period between September 16, 2019 to August 25, 2020. “Thinking about the coronavirus and its consequences” was first asked on March 24. *Questionnaire 0 is the recruitment questionnaire that the respondents fill in in the waiting area of the hospital. Participation in the study then continues via three questionnaires during pregnancy; questionnaires 1 to 3. The three health-related worry items had a “not applicable” response alternative that has been recoded to missing in this summary. The proportion that chose that option varied between 0.2 (*your own health*) and 3.8 percent (*your child's/children's health*). Note that the mean statistics are summaries of the stacked dataset, where the same person can be represented more than once, while the variables that are tabulated by categories are summaries of the non-stacked dataset.

Table S2. Robustness checks and model specification - Worry about your own health

|  | Model 1 |  | Model 2 |  | Model 3 |  | Model 4 |  | Model 5 |  |
| --- | --- | --- | --- | --- | --- | --- | --- | --- | --- | --- |
|  | Pooled OLS unw | | Pooled OLS | | Pooled OLS balanced | | Random effects | | Fixed effects | |
|  | *b* | *SE* | *b* | *SE* | *b* | *SE* | *b* | *SE* | *b* | *SE* |
| Post-March 12 | 0.098*** | 0.009 | 0.097*** | 0.009 | 0.103*** | 0.01 | 0.114*** | 0.008 | 0.139*** | 0.011 |
| Partner | -0.064*** | 0.009 | -0.066*** | 0.010 | -0.068*** | 0.011 | -0.059*** | 0.009 |  |  |
| Post-March 12 # Partner | -0.080*** | 0.013 | -0.080*** | 0.013 | -0.088*** | 0.015 | -0.089*** | 0.011 | -0.097*** | 0.014 |
| PW36 | -0.023*** | 0.006 | -0.025*** | 0.006 | -0.021*** | 0.006 | -0.026*** | 0.005 | -0.034*** | 0.006 |
|  |  |  |  |  |  |  |  |  |  |  |
| Constant | 0.369*** | 0.006 | 0.369*** | 0.006 | 0.361*** | 0.006 | 0.364*** | 0.006 | 0.332*** | 0.003 |
| R-squared within | 0.048 |  | 0.050 |  | 0.057 |  | 0.052 |  | 0.052 |  |
| R-squared between |  |  |  |  |  |  | 0.045 |  | 0.030 |  |
| R-squared overall |  |  |  |  |  |  | 0.048 |  | 0.035 |  |
| Sigma u |  |  |  |  |  |  | 0.204 |  | 0.252 |  |
| Sigma e |  |  |  |  |  |  | 0.184 |  | 0.184 |  |
| Rho |  |  |  |  |  |  | 0.550 |  | 0.652 |  |
| N Individual-wave dyads | 7,806 |  | 7,806 |  | 6,310 |  | 7,806 |  | 7,806 |  |
| N Individuals3 |  |  |  |  |  |  | 4,428 |  | 4,428 |  |

Notes: The table shows the output for a number of different model specifications in which the dependent variable is participants’ worry about their own health. This table serves as an illustration of the robustness of results across model specifications. The pooled OLS column, here called Model 2, reports the numbers for the questionnaire 1 in Figure 3. Note that the results are consistent across the various specifications. Model 1 is the same as Model 2, except that we do not use the nonresponse weight. Model 3 uses a subsample of the sample used in Model 2 that includes only the respondents who responded to all 3 questionnaires. Models 4 and 5 report the results from the random and fixed effects models, taking into account the multilevel structure of the panel.

+ p<0.10, * p<0.05, ** p<0.01, *** p<0.001. Reference category for post-March 12 is the period before March 12, and reference for partner respondent is pregnant woman. Reference category for is PW36 (pregnancy week 36, or “questionnaire 3”) is PW22-24 (pregnancy week 22-24, or “questionnaire 2”).

Figure S1. Institutional trust in healthcare (a) and politicians (b) as well as satisfaction with (c) planned prenatal and (d) emergency care during pregnancy


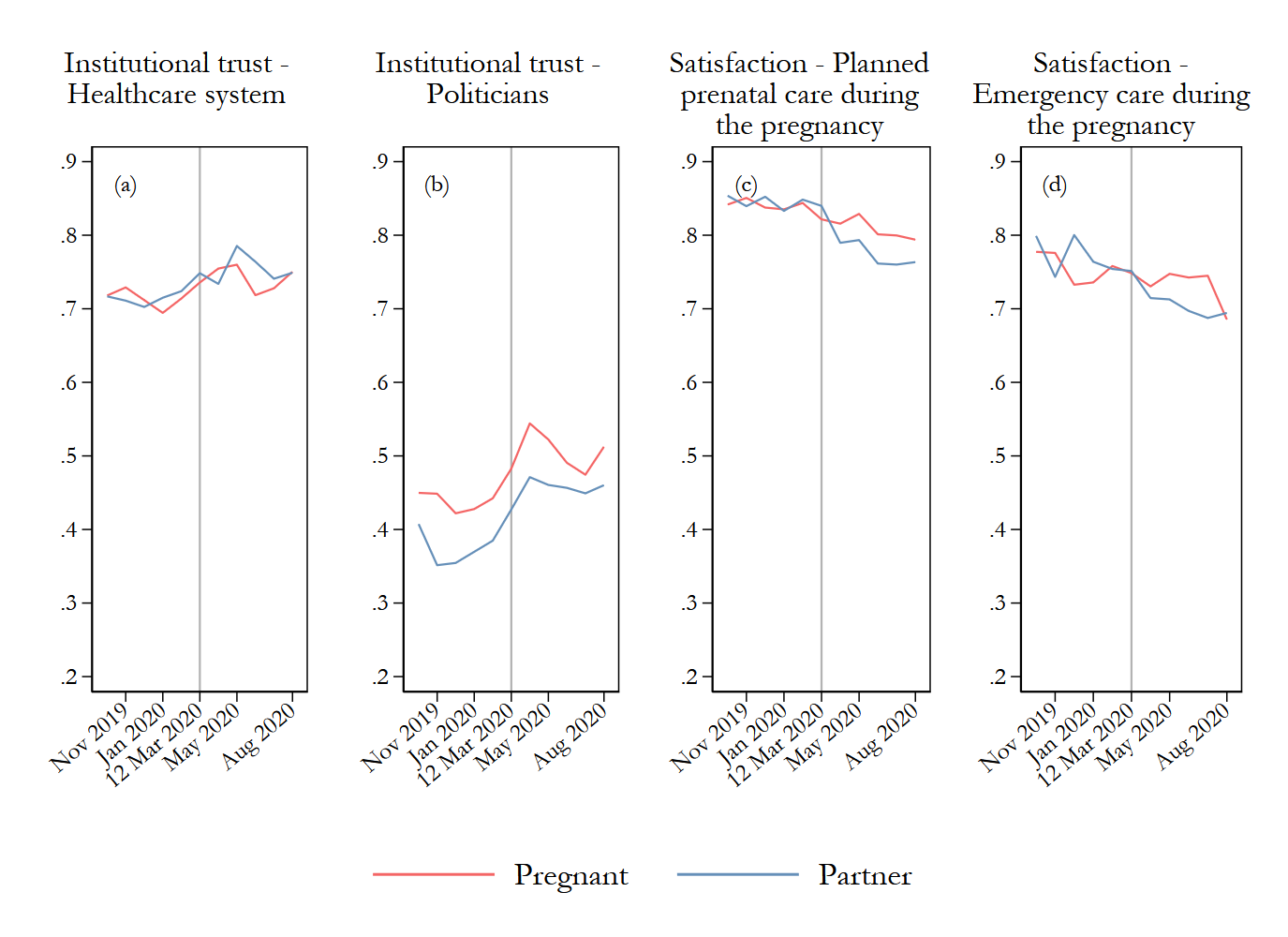


Notes: The increased levels of worry during the COVID-19 crisis shown in the article are not paralleled by a significant drop in trust in healthcare institutions and responsible politicians. Figure S1 shows that pregnant women and their partners express increased trust in healthcare (a) and politicians in the midst of the crisis (b). Figures (c) and (d) show high satisfaction with planned prenatal care and emergency care. However, there is a downward trend in both these measures during the pandemic. Monthly averages controlled for questionnaire fixed effects are reported. The wordings for the two questions regarding institutional trust are ‘*In general, how much confidence do you have in the following institutions and actors in Sweden?’*. The response options are ‘*1* *Very little’, ‘2 Not much’, ‘3 Neither a lot nor a little’, ‘4 Quite a lot’, ‘5 A lot’*. The wordings for the two questions regarding maternal care satisfaction are *‘How satisfied are you with the following types of care that you and your family received in connection with the pregnancy?’* The response options are ‘*1* *Very dissatisfied’, ‘2 Somewhat dissatisfied’, ‘3 Neither satisfied nor dissatisfied’, ‘4 Somewhat satisfied, ‘5 Very satisfied’, ‘6 Did not have any contact with these types of care’*. Participants answering the last alternative are excluded in this analysis.

Table S3. Overall contrasts of worry pre-/post-March 12

|  | **Contrast** | **SE** | **[95%Conf. Interval]** | | **df** | **F** | **P>F** | **N** | **R2** |
| --- | --- | --- | --- | --- | --- | --- | --- | --- | --- |
| **Post- vs pre-March 12** | | |  |  |  |  |  |  |  |
| Your own health | |  |  |  |  |  |  |  |  |
| Pregnant | 0.096 | 0.010 | 0.077 | 0.116 | 1 | 95.51 | 0.000 |  |  |
| Partner | 0.018 | 0.012 | -0.005 | 0.041 | 1 | 2.27 | 0.132 |  |  |
|  |  |  |  |  | 2 | 48.89 | 0.000 | 7,648 | 0.049 |
| Your child's/children's health | | |  |  |  |  |  |  |  |
| Pregnant | 0.072 | 0.011 | 0.051 | 0.093 | 1 | 45.34 | 0.000 |  |  |
| Partner | 0.063 | 0.014 | 0.035 | 0.090 | 1 | 19.54 | 0.000 |  |  |
|  |  |  |  |  | 2 | 32.44 | 0.000 | 7,253 | 0.0129 |
| Your partner's health | | |  |  |  |  |  |  |  |
| Pregnant | 0.089 | 0.010 | 0.069 | 0.110 | 1 | 73.19 | 0.000 |  |  |
| Partner | 0.074 | 0.013 | 0.049 | 0.100 | 1 | 32.77 | 0.000 |  |  |
|  |  |  |  |  | 2 | 52.98 | 0.000 | 7,577 | 0.0467 |

Notes: Numbers reported here tests the pre-/post-March 12 differences, i.e. the contrasts derived from pooled weighted OLS regressions. Each row first shows a contrast coefficient, a difference between the normalized averages pre- and post-March 12 (post-levels minus pre-levels). The *df*, *F* and *P*>*F* columns report *F*-tests of the contrasts, as well as a joint test.

Table S4. The American Association for Public Opinion Research (AAPOR) response rate statistics

|  | Pregnant |  |  |  |  | Partner |  |  |  |
| --- | --- | --- | --- | --- | --- | --- | --- | --- | --- |
| Questionnaire | 0 (PW12-19) | 1 (PW12-19) | 2 (PW22-24) | 3 (PW36) |  | 0 (PW12-19) | 1 (PW12-19) | 2 (PW22-24) | 3 (PW36) |
| No. of field days | 217 | 233 | 281 | 272 |  | 217 | 233 | 281 | 272 |
| Gross sample size (GSS)* | 6,133 | 3,827 | 3,035 | 3,340 |  | 6,045 | 3,113 | 2,243 | 2,644 |
| Completed responses** | 3,463 | 2,733 | 2,528 | 2,222 |  | 2,806 | 1,949 | 1,697 | 1,362 |
| Partials | 89 | 20 | 8 | 10 |  | 59 | 12 | 4 | 5 |
| Breakoffs | 276 | 6 | 2 | 0 |  | 248 | 7 | 4 | 2 |
|  |  |  |  |  |  |  |  |  |  |
| Recruitment rate (%)*** | 62.4 | *NA* | *NA* | *NA* |  | 51.5 | *NA* | *NA* | *NA* |
| Profile rate (%)† | 56.5 | *NA* | *NA* | *NA* |  | 46.4 | *NA* | *NA* | *NA* |
| Retention rate (%)†† | *NA* | 71.4 | 66.1 | 58.1 |  | *NA* | 62.6 | 54.5 | 43.8 |
|  |  |  |  |  |  |  |  |  |  |
| AAPOR RR5 (%)‡ | *NA* | 71.4 | 83.3 | 66.5 |  | *NA* | 62.6 | 75.7 | 51.5 |
| AAPOR RR6 (%) | *NA* | 71.9 | 83.6 | 66.8 |  | *NA* | 63.0 | 75.8 | 51.7 |

Notes: PW = pregnancy week. * For time point 0, the GSS is an estimation of the total population sample. It consists of the number of pregnant women with an ultrasound appointment at the Östra Sahlgrenska University Hospital during the recruitment period, and the estimated number of partners, which is based on the number of consents given at recruitment. The estimated number of partners was calculated based on the share of women in the profile survey who stated that they did not have a partner. For questionnaires 1–3, the GSS is based on the number of individuals who received the questionnaire. ** The American Association for Public Opinion Research (AAPOR) provides a set of guidelines for survey response rates in the *Standard definitions* (2016) report. By this definition, to count as a completed survey response, more than 80 percent of the questions have to be filled out, partial responses have between 50 and 80 percent non-missing answers, while breakoffs are below 50 percent non-missing answers. ***An individual is regarded as recruited when we received a valid email address in the recruitment survey (questionnaire 0) as well as registered consent to participate in the Swedish Pregnancy Panel. † Profile rate is the number of fully completed recruitment surveys divided by the estimated population. †† The retention rate of the panel indicates the proportion of respondents that are still answering a given questionnaire. This is calculated by dividing the number of completed responses for each questionnaire by the number of recruited respondents to the Swedish Pregnancy Panel who were eligible to answer that specific questionnaire before August 25, 2020. Eligibility was based on pregnancy week only. ‡ AAPOR response rate 5 (RR5) is the number of completed responses as a proportion of the invited sample; RR6 is the number of completed *and* *partial* responses as a proportion of the invited sample.

As a result of panel attrition, the distribution of participants differs somewhat between questionnaires. Most notably, partners drop out more often than pregnant women, as do participants with low education as compared to those with high education. The proportion pregnant respondents increases from 55 percent (in the recruitment questionnaire), to 58, 60 and 62 percent respectively in questionnaires 1, 2 and 3. The proportion of participants with low education (i.e. those with high school education and below) decreases from 21 percent in the recruitment questionnaire to 19.4, 19.3 and 17 percent in the following questionnaires. There is also a higher attrition among younger participants in the panel. 21-25 year-olds constitute 7 percent in the recruitment questionnaire, and 6, 5, 4 percent receptively in the surveys that follow thereafter.
